# Modulating phenylalanine metabolism by *L. acidophilus* alleviates alcohol-related liver disease through enhancing intestinal barrier function

**DOI:** 10.1186/s13578-023-00974-z

**Published:** 2023-02-04

**Authors:** Liuying Chen, Pengcheng Yang, Lilin Hu, Ling Yang, Huikuan Chu, Xiaohua Hou

**Affiliations:** Division of Gastroenterology, Union Hospital, Tongji Medical College, Huazhong University of Science and Technology, 1277 Jiefang Avenue, Wuhan, 430022 China

**Keywords:** Alcohol-related liver diseases, Phenylalanine metabolism, *Lactobacillus acidophilus*

## Abstract

**Background:**

Impaired metabolic functions of gut microbiota have been demonstrated in alcohol-related liver disease (ALD), but little is known about changes in phenylalanine metabolism.

**Methods:**

Bacterial genomics and fecal metabolomics analysis were used to recognize the changes of phenylalanine metabolism and its relationship with intestinal flora. Intestinal barrier function was detected by intestinal alkaline phosphatase (IAP) activity, levels of tight junction protein expression, colonic inflammation and levels of serum LPS. *Lactobacillus acidophilus* was chosen to correct phenylalanine metabolism of ALD mice by redundancy analysis and Pearson correlation analysis.

**Results:**

Using 16S rRNA sequencing and ultra-performance liquid chromatography-tandem mass spectrometry (UPLC-MS/MS) methods, we identified elevated levels of phenylalanine and its’ metabolites in the gut of alcohol-fed mice compared to control mice and were negatively correlated with the abundance of *Lactobacillus*, which mainly metabolized phenylalanine. The intestinal phenylalanine level was positively correlated with the colon inflammatory factors TNF-α and IL-6, and negatively correlated with ZO-1 and Occludin. While intestinal alkaline phosphatase (IAP) activity was negatively correlated with the colon inflammatory factors TNF-α, IL-6 and MCP-1, and positively correlated with ZO-1 and Occludin. Increased phenylalanine inhibited IAP activity, blocked LPS dephosphorylation, increased colonic inflammation and bacterial translocation. Phenylalanine supplementation aggravated alcohol-induced liver injury and intestinal barrier dysfunction. Among the 37 *Lactobacillus* species, the abundance of *Lactobacillus acidophilus* was most significantly decreased in ALD mice. Supplementation with *L. acidophilus* recovered phenylalanine metabolism and protected mice from alcohol-induced steatohepatitis.

**Conclusions:**

Recovery of phenylalanine metabolism through the oral supplementation of *L. acidophilus* boosted intestinal barrier integrity and ameliorated experimental ALD.

**Supplementary Information:**

The online version contains supplementary material available at 10.1186/s13578-023-00974-z.

## Background

Gut dysbiosis has been shown to participate in the progression of alcohol-related liver diseases (ALD). Germ-free mice exposed to drinking water supplemented with alcohol for 7 days did not have liver injury or hepatic inflammation compared with conventional mice [1]. Germ-free mice transplanted with the intestinal microbiota of patients with severe alcoholic hepatitis had a more severe liver injury and inflammation and increased intestinal permeability than those transplanted with the intestinal microbiota of patients with no alcoholic hepatitis [2]. Alcohol administration reduced bacterial diversity and uniquely shaped the composition of the intestinal bacteria. Alcohol-dependent individuals showed a decrease in the species of *Bifidobacterium* and an increase in the genera of *Dorea*, *Blautia* and *Megasphaera* compared with controls [3]. Patients with alcoholic hepatitis showed a significant increase in the abundance of *Enterococcus* species in feces, compared with controls [4]. Consistent with previous studies [5–7], we also found alcohol-treated mice had reduced abundances of the *Lactobacillus* and *Akkermansia* genera, compared with pair-fed mice.

Alcohol-induced functional changes in the intestinal microbiota are also important for the pathogenesis of alcoholic liver disease. Short-chain fatty acids (SCFAs), which are by-products of fermented fiber by intestinal bacteria, showed a significant decrease in the colon of rats chronically exposed to alcohol, compared with control rats [8]. Supplementation of tributyrin and propionate improved the intestinal barrier function and alleviated liver injury in the chronic-binge alcohol-fed mice [9, 10]. Chronic administration of ethanol inhibited bacterial gene expression involved in the biosynthesis of saturated fatty acids, compared to controls, and dietary supplementation of saturated long chain fatty acids prevented alcohol-induced gut leakiness and liver damage [7]. Chronic-binge alcohol treatment inhibited bacterial catabolism of tryptophan into indoles, reduced REG3G expression in enterocytes and increased intestinal bacterial translocation [11]. Serum concentrations of tryptophan metabolites decreased in patients with severe alcoholic hepatitis compared to alcoholic patients with hepatitis [12]. Although the role of altered aromatic amino acid metabolism has been demonstrated in alcohol-induced liver damage, little is known about changes in phenylalanine metabolism.

In our previous study, we found that intestinal L-phenylalanine levels increased significantly in alcohol-fed mice, compared to control mice. Similar findings have reported that chronic alcohol consumption increases the level of L- phenylalanine in the feces of mice [13]. Liver and serum levels of phenylalanine were found to be elevated in patients with alcohol-associated liver damage [14, 15], but decreased in the feces of patients with actively drinking cirrhosis [16]. L-phenylalanine has been proven to act as a selective inhibitor of epithelial alkaline phosphatase [17]. Intestinal alkaline phosphatase (IAP) has shown many physiological functions, including reduced endotoxemia and subsequent inflammation, and maintenance of intestinal barrier function [18]. Aging mice that showed a decreased in IAP activity had high intestinal permeability and systemic inflammation [19]. Oral IAP supplementation improved intestinal barrier function, attenuated alcohol-induced liver steatosis and inflammation [20], and rescued bile duct ligation, and carbon tetrachloride-4 (CCl4)-induced liver fibrosis [21]. In addition to the inhibitory effect of phenylalanine on IAP, its metabolites produced by intestinal bacteria also act as modulators of gastrointestinal function. In this study, we examined the role of alcohol-induced abnormal phenylalanine metabolism in intestinal barrier injury and liver injury. We also investigated how *Lactobacillus acidophilus* supplementation restored phenylalanine metabolism and offered protection from ethanol-induced hepatic steatosis.

## Methods

### Animal experiments

Eight weeks-old male C57BL/6 mice were purchased from Vital River Labs (Beijing, China). A Lieber-De Carli diet was used to build an acute-on chronic alcohol exposure model based on the results of a previous study [22]. Briefly, the mice were first acclimated to a liquid diet for 5 days, then fed on a Lieber-De Carli diet containing 5 vol% ethanol for 10 days. Finally, they were binged with 5 g of alcohol per kilogram of body weight. The control group of mice was fed maltose dextrin containing the same calories instead of ethanol. Tissues and serum were collected 9 h after the binge for follow-up experiments.

For phenylalanine supplementation, mice fed ethanol and isocaloric were gavaged daily with 200 mg/Kg phenylalanine or PBS for a total of 15 days.

For *Lactobacillus* treatment, mice fed ethanol and isocaloric were gavaged daily with *L. acidophilus* (5 × 10^8^ colony-forming units (CFU) in 200 μl phosphate-buffered saline (PBS)) or PBS for a total of 15 days. *L. acidophilus* (ATCC 43,550) was purchased from the Shanghai Conservation Biotechnology Center and incubated in the MRS broth medium at 37 ℃.

The animal studies complied with the Animal Research: Reporting of In Vivo Experiments (ARRIVE) guidelines.

### 16S rRNA sequencing

The caecal stool was collected and DNA was extracted from the ethanol-fed and isocaloric-fed mice with and without *L. acidophilus* treatment. The V3-V4 hypervariable regions of 16S rRNA were amplified. Sequencing libraries were generated using NEB Next^®^ Ultra DNA Library Prep Kit (Illumina, USA). At last, the library was sequenced on an Illumina NovaSeq platform and 250 bp paired-end reads were generated. QIIME software was used to construct the operational taxonomic unit (OUT) abundance tables of each sample. The alpha diversity of the intestinal microbiota was measured using the Shannon and Chao indexes, and the beta diversity of the intestinal microbiota was analyzed by principal coordinate analysis (PCoA) and the partial least squares discriminant analysis (PLS-DA). The linear discriminant analysis effect size (LEfSe) was applied to identify species with significant differences in abundance between the groups using the Wilcoxon rank sum test. Comparison with sequencing data on known metagenomic functional data was performed using PICRUSt software, while KEGG metabolic pathways were used to predict differences in functional genes of microbial communities between the groups. To identify bacteria species associated with phenylalanine metabolism, redundancy analysis (RDA) were performed.

### Untargeted metabolome analysis

Fecal samples were obtained from the ethanol-fed and isocaloric-fed mice with and without *L. acidophilus* treatment and analyzed using UPLC-MS/MS at positive ion mode electrospray ionization (ESI) and negative ion mode ESI. Raw data were processed for quality control and normalization, and then the PLS-DA model was used to discriminate significant differences between the groups. The differences in metabolite levels between the groups were compared using the Student's t test.

### Measurement of intestinal alkaline phosphatase (IAP) and LPS dephosphorylation activities

The modified Gomori calcium-cobalt method was used to measure IAP activity. According to the instructions provided with the kit (Solarbio, Beijing, China), paraffin-embedded colon tissues were sliced and dewaxed. The samples were then incubated with sodium glycerol phosphate solution or LPS solution (1 mg/ml) for 4 h, and cobalt buffer for 5 min at 37 ℃. Hydrogen sulfide working buffer was added to the samples and allowed to react for 2 min, and then rinsed with distilled water. Finally, nuclear fast red was used to visualize the nuclei.

### Liver histopathology

Liver tissues were stained with hematoxylin eosin (HE) to observe signs of liver injury. To assess liver steatosis, liver tissues were embedded in OCT, and cut to a thickness of 10 μm. The frozen sections were then stained with oil red O (Sigma-Aldrich, Shanghai, China).

### Serum ALT, AST, and LPS detection

Serum levels of alanine aminotransferase (ALT) and aspartate aminotransferase (AST) were measured using commercial kits (Nanjing Jiancheng Institute of Bioengineering, Nanjing, China). Endotoxin levels were examined using a LPS ELISA kit (Solarbio, Beijing, China) according to the protocols suggested by the manufacturer.

### Immunohistochemistry (IHC) and immunofluorescence (IF)

Immunohistochemical staining was performed using a goat anti-rabbit IgG (H + L) IHC kit (Servicebio, Wuhan, China). Colon slices were incubated with F4/80 (1:300, Servicebio), then stained with a DAB working solution, while the nucleoli were stained with hematoxylin. For IF staining, colon tissues were incubated with LPS (1:50, Hycult Biotech, Netherlands), ZO-1 (1:200, Servicebio, Wuhan, China), and Occludin (1:200, Proteintech, Chicago, United States) antibodies. The slices were then stained with the Alexa Fluor 594 conjugated goat anti-rabbit IgG (H + L) secondary antibody (1:500, CST, Massachusetts, USA), and Alexa Fluor 594 conjugated goat anti-Mouse IgG (H + L) secondary antibody (1:500, CST). The nuclei were stained with DAPI.

### Reverse transcription-polymerase chain reaction (RT-PCR)

Total RNA was extracted from colon tissues of ethanol-fed and control mice with and without *L. acidophilus* supplementation using TRIzol reagent (Invitrogen, California, USA). Total RNA was then reverse transscribed using the PrimeScript™ RT Master Mix (RR036A, TaKaRa). PCR amplifications were performed on a LightCycler 96 amplifier using the PowerUp™ SYBR™ Green Master Mix (Invitrogen, Carlsbad, CA). The detection of liver translocation bacteria was carried out using previously published methods [11, 23]. The primer sequences used are shown in Additional file 1: Table S1.

### Cell culture and treatment

Primary hepatocytes were isolated from C57BL/6 mice according to previous study [24]. Starved hepatocytes (3% fetal bovine serum) were stimulated with ethanol (50 mM) and phenylalanine (100 μM) for 24 h. Hepatocyte cytotoxicity was measured using the lactate dehydrogenase (LDH) cytotoxicity assay kit (Beyotime Biotechnology, Shanghai, China).

Human colon epithelial cells (NCM460) were cultured in DMEM medium with 10% fetal bovine serum. Then NCM460 cells were starved with 3% fetal bovine serum and treated with ethanol (50 mM) and phenylalanine (100 μM) for 24 h. The IAP expression was measured by immunofluorescence (1:300, Servicebio, Wuhan, China), and ZO-1 and Occludin expressions were measured by western blotting.

### Fecal lactobacillus species identification

A total of 37 *lactobacillus* species were identified and compared in fecal samples obtained from ethanol-fed and isocaloric-fed mice, based on a previous study [25]. Total fecal DNA was extracted using a fecal genomic DNA extraction kit (TIANGEN, Beijing, China). Species-specific primer sequences and PCR amplification conditions used in previous study were used [25].

### In vivo intestinal permeability assay

Intestinal permeability was assessed by gastric gavage of fluorescein isothiocyanate-dextran (FITC-dextran) (4 kDa; Sigma-Aldrich). Mice were gavaged with 200 μL of FITC-dextran (100 mg/mL) 5 h after ethanol binge, and the blood samples were collected 4 h later. Serum FITC-dextran levels were measured using a fluorimeter.

### Statistics

Data were shown as mean ± SEM. Student’s t test and Mann-Whitney test were used to analyze the differences between groups. P value of less than 0.05 was considered to indicate significance.

## Results

### Phenylalanine metabolism by intestinal bacteria was disordered in the ethanol-fed mice

**Fig. 1:**
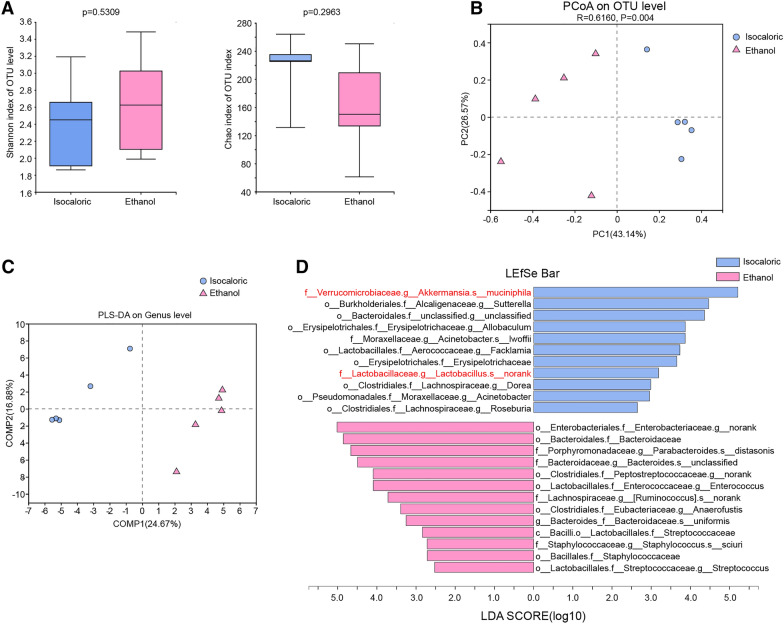
Chronic and binge ethanol feeding caused gut dysbiosis of mice. **A** Alpha diversities of intestinal bacteria measured by shannon and chao indexes of Chronic–binge ethanol feeding mice and isocaloric feeding mice. **B**, **C** The principal coordinate analysis (PCoA) (**B**) and partial least squares discriminant analysis (PLS-DA) (**C**) revealed the beta diversities of intestinal bacteria. **D** Linear discriminant analysis Effect Size (LEfSe) analysis detected taxa with significant differences between alcohol exposed and control groups

The alpha diversities of intestinal bacteria determined using the Shannon and Chao indices were not significantly different between the chronic–binge ethanol-fed mice and the control mice (Fig. 1A). However, the principal coordinate analysis (PCoA) and the partial least squares discriminant analysis (PLS-DA) revealed that the bacterial composition was significantly different between the mice fed ethanol and the control mice (Fig. 1B, C). Consistent with the results of previous studies [6], the abundance of *Akkermansia muciniphila* decreased significantly in the chronic–binge ethanol-fed mice (Fig. 1D). We also observed that the *Lactobacillus* genus showed a prominent decline.

**Fig. 2:**
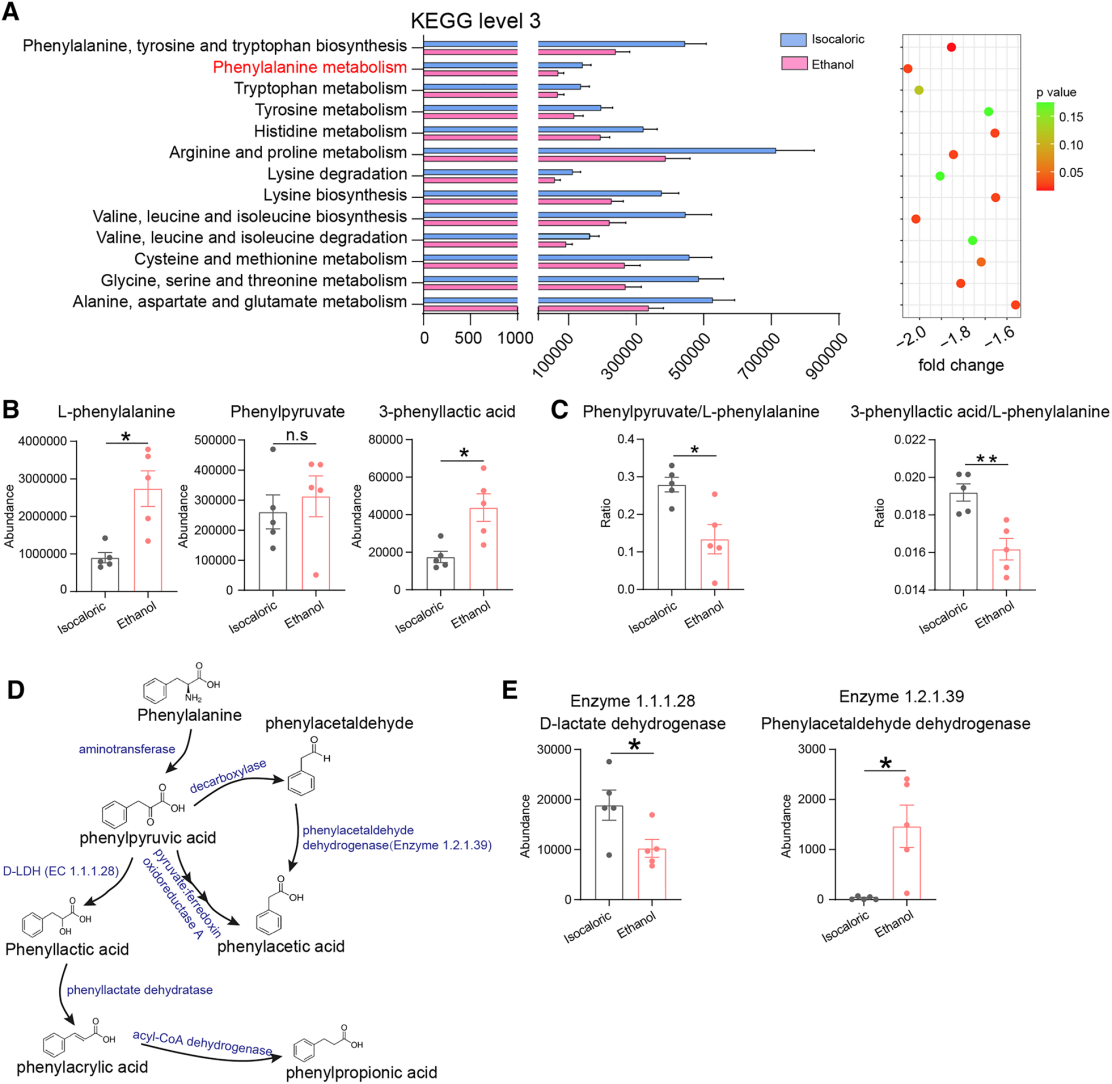
Phenylalanine metabolism was destroyed in alcohol administration mice. **A** Comparison of KEEG module abundances of amino acid metabolism that predicted by PICRUSt using 16S data between alcohol exposed and control groups. **B** Comparison of the abundance of phenylalanine and its’ metabolites phenylpyruvate and 3-phenyllactic acid in Chronic-binge ethanol feeding mice and control mice. **C** Comparison of the ratios of phenylpyruvate to phenylalanine and 3-Phenyllactic acid to phenylalanine between the above groups. **D** The pathway of phenylalanine catabolism by gut microbes (refered to Martin F. Laursen et al. [27] and Dylan Dodd et al. [28]). **E** Abundances of Bacterial phenylalanine metabolizing enzymes D-lactate dehydrogenase and phenylacetaldehyde dehydrogenase predicted by PICRUSt method in the above groups. n.s, no significant. *p value < 0.05. **p value < 0.01

To explore the functions of intestinal bacteria, we performed a KEGG pathway analysis. Among the changes in amino acid metabolism, which were the focus of this study, a decrease in phenylalanine metabolism of ethanol feeding mice caught our attention (Fig. 2A). This result was supported by our previous findings on elevated phenylalanine levels in the gut of alcohol-fed mice, compared to control mice (Fig. 2B). Two metabolites were detected in the fecal matter of the ethanol-fed and control mice. The 3-phenyllactic acid level increased in the ethanol-fed mice in contrast to the control mice, but the phenylpyruvate level did not show significant differences (Fig. 2B). The ratios of phenylpyruvate to L-phenylalanine and 3-phenyllactic acid to L-phenylalanine were lower in the alcohol-fed mice than in the control mice (Fig. 2C). The abundance of bacterial D-lactate dehydrogenase (D-LDH), which has been reported to metabolize phenylpyruvic acid into phenyllactic acid [26–28], was lower in the alcohol-fed mice than in the control mice (Fig. 2D, E). The level of bacterial phenylacetaldehyde dehydrogenase was higher in alcohol-fed mice than in control mice.

### High levels of phenylalanine inhibited intestinal alkaline phosphatase activity (IAP) and correlated with the colonic inflammatory injury of ethanol feeding mice

**Fig. 3:**
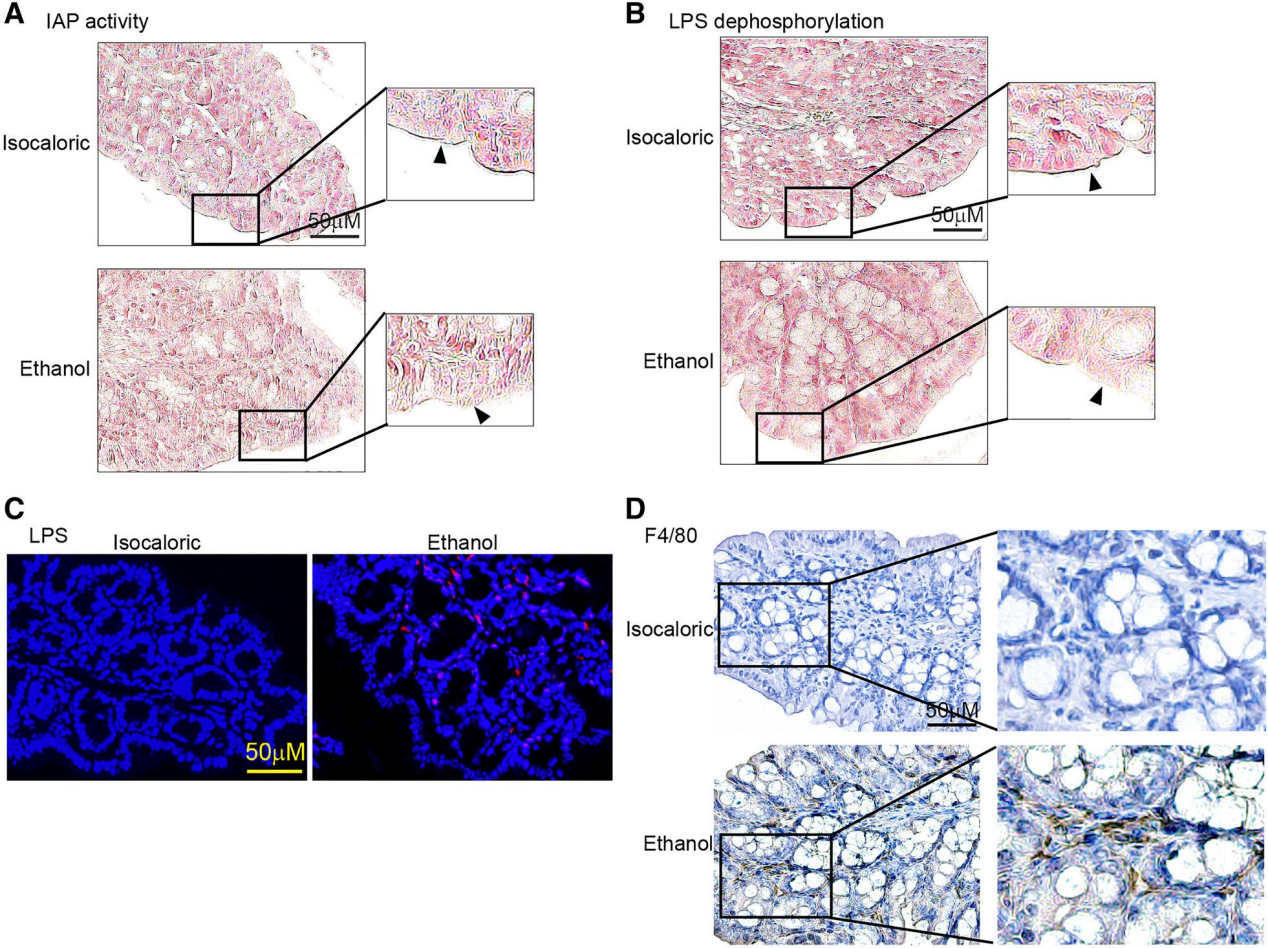
High level of phenylalanine inhibited IAP activity and increased colonic inflammatory reaction. **A**, **B** IAP activity (**A**) and LPS dephosphorylation (**B**) measured by Modified Gomori calcium-cobalt method. **C** Colonic bacteria invasion of chronic and binge ethanol-fed mice and isocaloric-fed mice by LPS immunofluorescence staining. **D** Colonic macrophages invasion of chronic and binge ethanol-fed mice and Isocaloric-fed mice by F4/80 immunofluorescence staining

IAP expressed on the apical surfaces of enterocytes plays an important role in detoxifying LPS and protecting the intestinal barrier [29]. L-phenylalanine is known as an inhibitor of IAP [17]. Then, we measured the activity of IAP in the chronic–binge ethanol-fed mice and control mice. As shown in Fig. 3A, colonic IAP activity had decreased significantly in chronic–binge ethanol-fed mice compared to control mice. The ability of IAP to dephosphorylate and inactivate LPS also decreased in the alcohol-fed mice (Fig. 3B). Consistently, colonic bacterial invasion detected by immunofluorescence with anti-LSP antibody increased in mice fed alcohol, compared to control mice (Fig. 3C). Furthermore, an increase in pro-inflammatory macrophage infiltration into the colon was observed in alcohol-fed mice, compared to controls (Fig. 3D, Additional file 1: Figure S1).

To determine whether the increase in colonic inflammatory reaction was related to the high level of phenylalanine and decreased IAP activity, we analyzed the correlation of intestinal phenylalanine content and IAP activity, with the expression levels of inflammatory factors and tight junction proteins. The level of phenylalanine was positively correlated with the mRNA levels of TNF-α, and IL-6, and negatively correlated with the mRNA levels of ZO-1 and Occludin (Tables 1 and 2). While IAP activity was negatively correlated with the mRNA levels of TNF-α, IL-1, MCP-1, and positively correlated with the mRNA levels of ZO-1 and Occludin (Tables 1 and 2).

**Table 1:** Correlation of L-phenylalanine and IAP activity with the mRNA expressions of colonic inflammatory factors

	L-phenylalanine		IAP activity*	
	Pearson r	P value	Pearson r	P value
TNF-α	0.7736	0.0087	− 0.7191	0.0191
IL-6	0.7917	0.0064	− 0.6076	0.0624
IL-1	0.4608	0.1801	− 0.7657	0.0098
TGF-β	0.04465	0.9025	− 0.4283	0.2168
MCP-1	0.3221	0.3641	− 0.7487	0.0127
IFN-γ	0.6195	0.0561	− 0.5744	0.0824

^*^measured by positive staining coverage of colonic epithelial cells

**Table 2:** Correlation of L-phenylalanine and IAP activity with the mRNA expressions of tight junction proteins

	L-phenylalanine		IAP activity*	
	Pearson r	P value	Pearson r	P value
ZO-1	− 0.6968	0.0251	0.6887	0.0276
Occludin	− 0.7279	0.017	0.7100	0.0214

^*^measured by positive staining coverage of colonic epithelial cells

### Phenylalanine supplementation aggravated alcohol-induced liver injury and intestinal barrier dysfunction

**Fig. 4:**
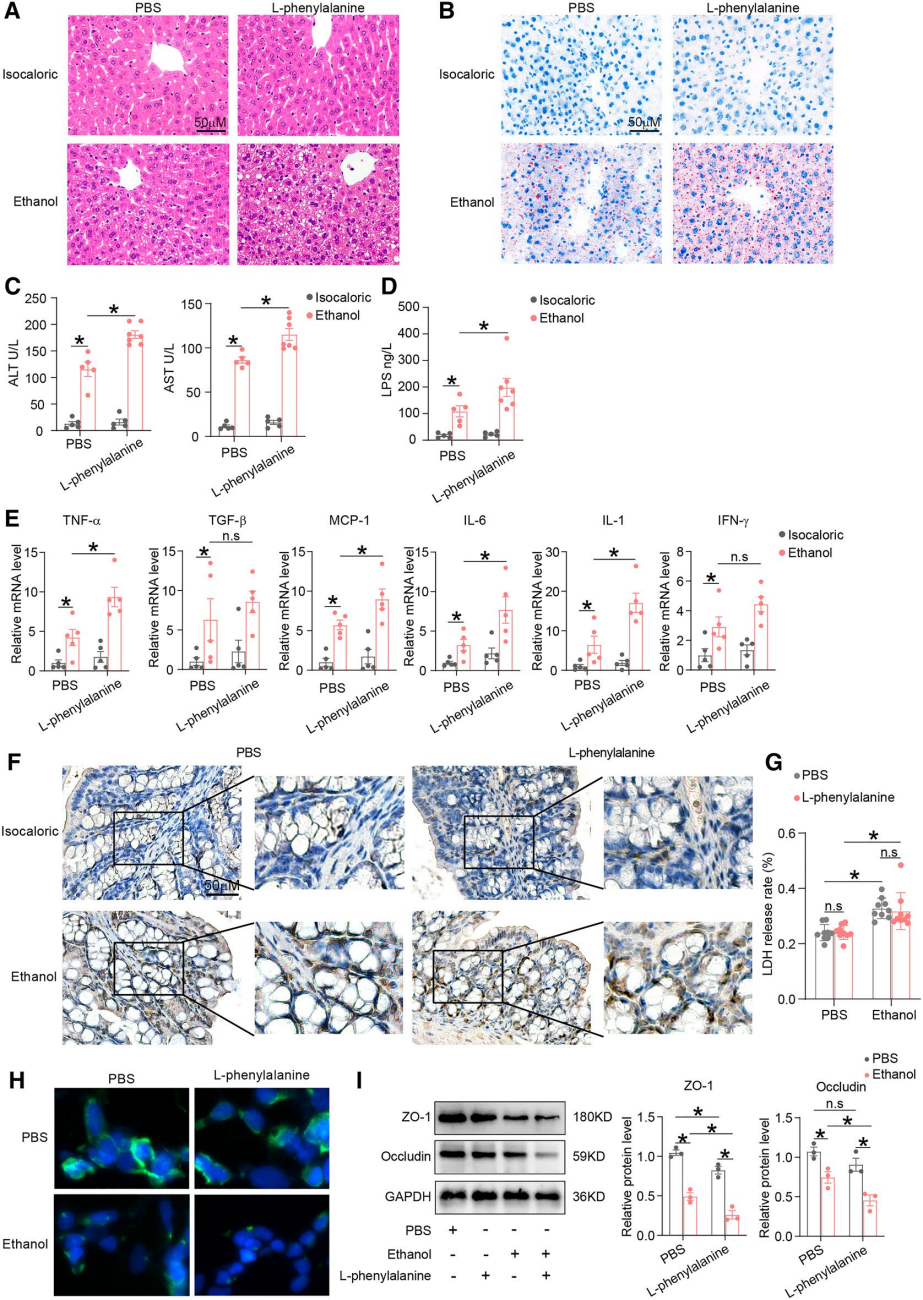
Phenylalanine supplementation worsened alcohol-induced liver injury and intestinal barrier dysfunction. **A** HE staining of liver tissues of alcohol-fed and isocaloric-fed mice orally treated with phenylalanine or PBS. **B** Evaluation of hepatic steatosis by Oil-red staining of above groups. **C**, **D** Serum ALT, AST (**C**) and LPS (**D**) levels of above mice. **E** Inflammatory cytokines expressions of colonic tissues measured by qPCR of above mice. **F** Macrophage invasion of colonic tissues measured by IHC of above mice. **G** Cytotoxic of primary hepatocytes treated with ethanol or phenylalanine measured by LDH release rate. **H** IAP expression of colonic epithelial cells with ethanol or phenylalanine measured by IF. **I** ZO-1 and Occludin expressions of above cells measured by western blotting. *p value < 0.05. n.s, no significant

To further evaluate whether phenylalanine was responsible for alcohol-related intestinal barrier function impairment, alcohol-fed and isocaloric-fed mice were treated with phenylalanine. As shown in Fig. 4A–C, mice with phenylalanine supplementation had more severe alcohol-induced liver injury and steatosis. Intestinal permeability measured by level of serum LPS was increased in alcohol-exposed mice supplemented with phenylalanine, compared to mice supplemented with PBS (Fig. 4D). In addition, phenylalanine is associated with increased colonic inflammation. TNF-α, MCP-1, IL-6 and IL-1 expressions, and macrophage infiltration were increased in alcohol plus phenylalanine treated mice, as compared to alcohol plus PBS treated mice (Fig. 4E, F).

In vitro, primary mouse hepatocytes were isolated and treated with alcohol or phenylalanine. Phenylalanine did not affect hepatocytes cytotoxicity caused by alcohol (Fig. 4G). Phenylalanine intervention exacerbated reduction of alcohol-induced IAP expression of human colon epithelial cells (NCM460) (Fig. 4H). The expressions of tight junction proteins ZO-1 and Occludin were also decreased in ethanol plus phenylalanine treated NCM460 cells compared to ethanol plus PBS treated NCM460 cells (Fig. 4I).

### Decreased abundance of *Lactobacillus* related with abnormal metabolism of phenylalanine

**Fig. 5:**
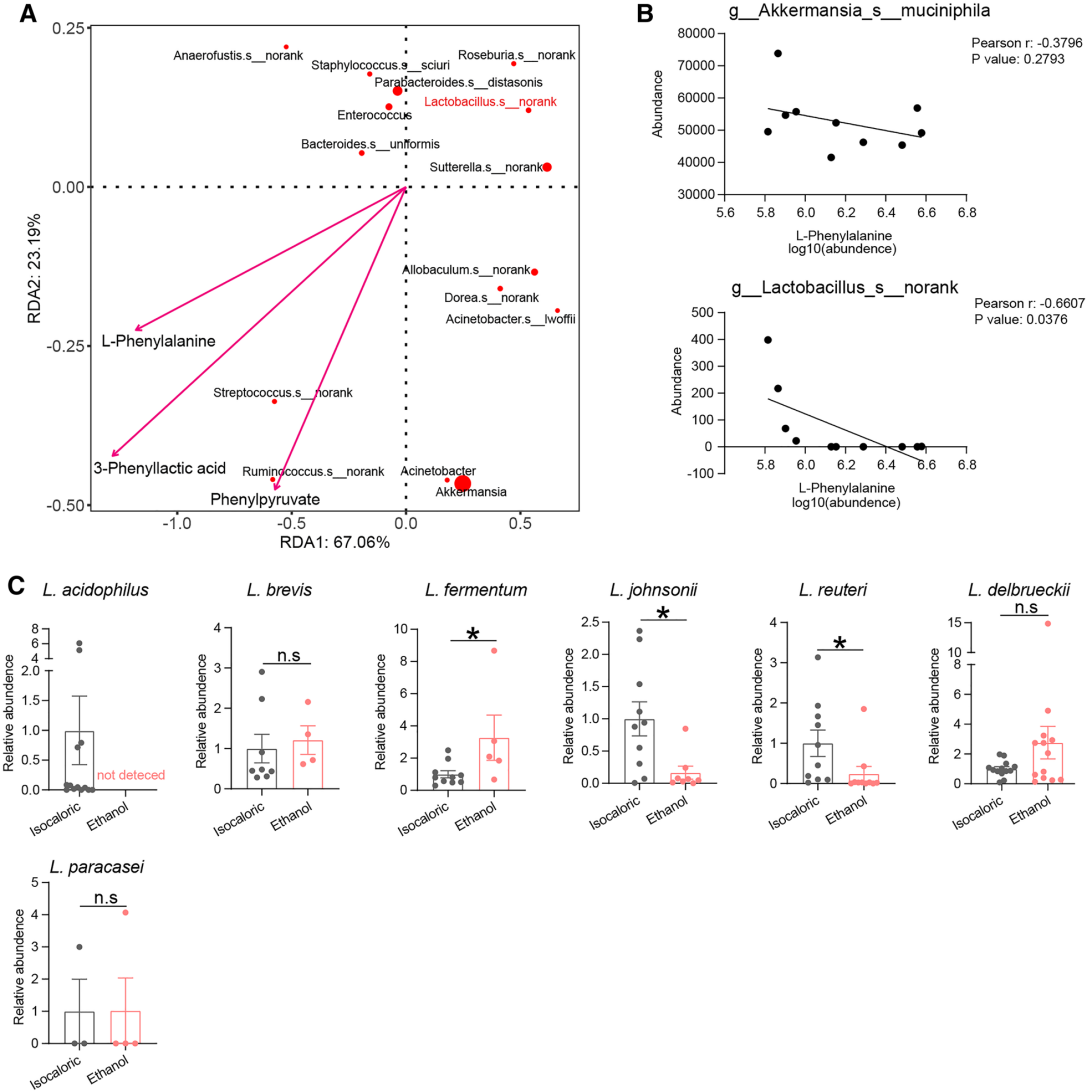
Decreased abundance of *Lactobacillus* was related to high level of intestinal phenylalanine. **A** Redundancy analysis (RDA) of species that different between alcohol-fed and control mice, and phenylalanine metabolites. **B** Correlation analysis of *Akkermansia*, *Lactobacillus* and intestinal levels of phenylalanine. **C** Abundances of *Lactobacillus genera* in Chronic-binge ethanol feeding mice and control mice measured by PCR. not detected, defined as CT > 40. n.s, no significant. *p value < 0.05

Redundancy analysis (RDA) using bacteria species that different between alcohol-fed and control mice, and phenylalanine and its’ metabolites, showed only *Lactobacillus* was obviously negative related with phenylalanine and its metabolites phenylpyruvate and 3-phenyllactic acid (Fig. 5A). Although alcohol exposure can cause a greater depletion of *Akkermansia* than *Lactobacillus*, only *Lactobacillus* was positively correlated with intestinal phenylalanine activity (Fig. 5B). To further identify the *Lactobacillus* species, which was affected by alcohol feed, 37 species-specific primer pairs were selected and used to differentiate species using PCR assays based on methods previously described [25]. Seven *Lactobacillus* species were detected in at least four samples from mice fed ethanol (Additional file 1: Table S2, Fig. 5C). The alcohol-fed mice showed a decrease in *L. reuteri*, *L. johnsonii,* and *L. acidophilus,* compared with the control mice, especially *L. acidophilus,* since it was not detected in alcohol-fed mice (CT value > 40). *L. fermentum* abundance was higher in alcohol-fed mice, compared to control mice. On the contrary, there were no significant differences in *L. paracasei*, *L. delbrueckii,* and *L. brevis* abundances between the alcohol-fed mice and the control mice.

### Supplementation with *L. acidophilus* alleviated ethanol-induced liver injury and intestinal barrier damage

**Fig. 6:**
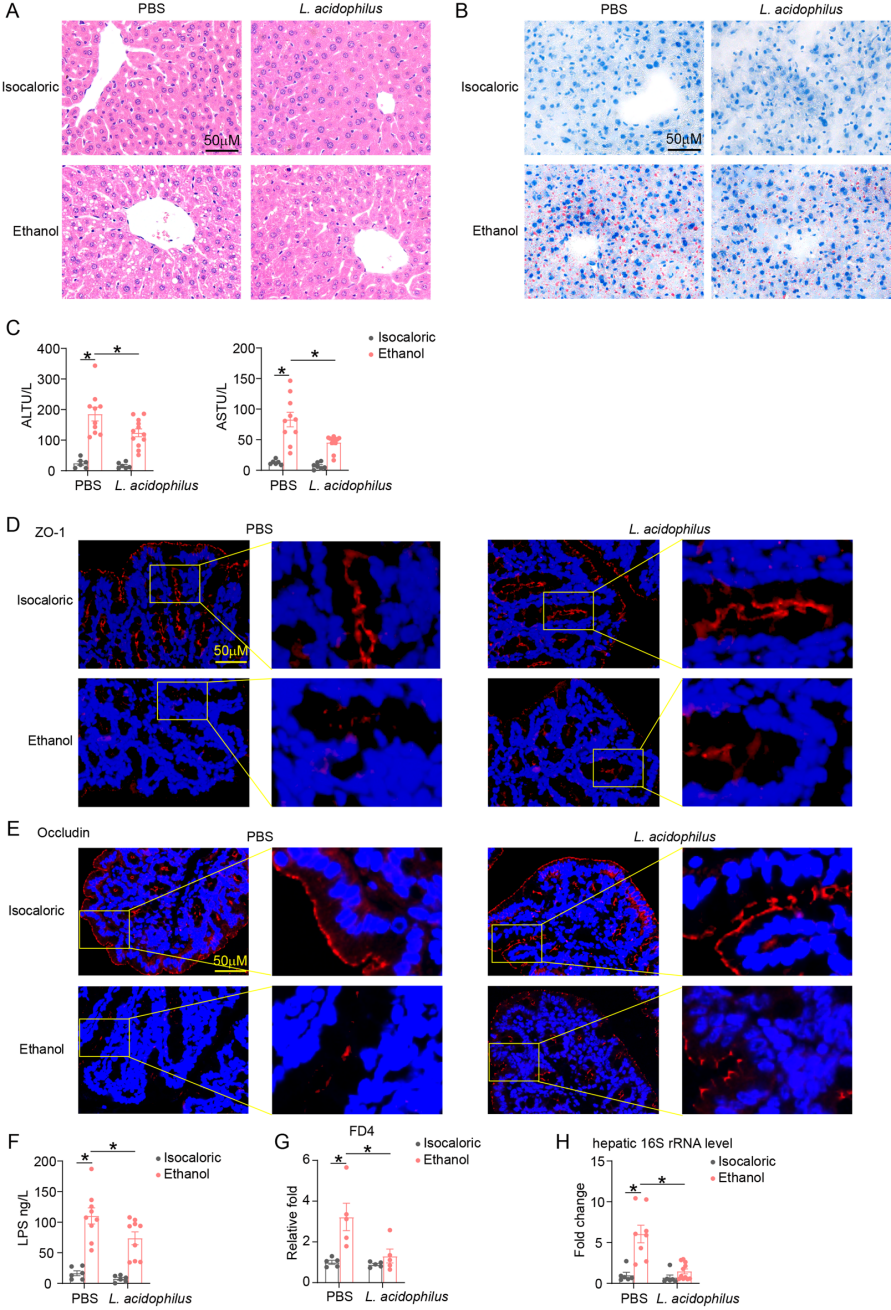
Supplementation with *L. acidophilus* alleviated ethanol-induced liver injury and restored intestinal barrier function. **A** HE staining of liver tissues of chronic and binge ethanol-fed mice and isocaloric-fed mice with and without *L. acidophilus* supplementation. **B** Assessment of hepatic steatosis with Oil-red staining. **C** Serum ALT and AST levels of ALD mice with and without *L. acidophilus* treatment. **D**, **E** Colonic ZO-1 (**D**) and Occludin (**E**) immunofluorescence analysis of above groups. **F**, **G** In vivo evaluating gut permeability of above groups of mice by serum LPS (**F**) and FD4 (**G**) levels. (**H**) hepatic 16S rRNA expression determined by PCR to detect bacterial translocation of above groups. *p value < 0.05

Since *L. acidophilus* has been shown to express D-LDH [30] and is one of the main bacteria metabolizing phenylalanine [31], we hypothesized that *L. acidophilus* administration may ameliorate alcohol-induced liver injury. Oral *L. acidophilus* supplementation attenuated liver steatosis and liver damage, as demonstrated by the significant reduction in serum levels of ALT and AST and the accumulation of liver lipid droplets (Fig. 6A–C). IF staining of ZO-1 and Occludin revealed that chronic-binge ethanol feeding injured the colonic barrier of the mice, and *L. acidophilus* supplementation reduced damage of the intestinal barrier function (Fig. 6D, E). Consistent with the expression of tight junction proteins, an increase in serum levels of LPS, FITC labeled dextran (4KD), and liver bacteria was found in alcohol-exposed mice compared to control mice, and decreased after *L. acidophilus* supplementation (Fig. 6F–H).

### Supplementation with *L. acidophilus* ameliorated ethanol-induced inflammatory injury of colon

**Fig. 7:**
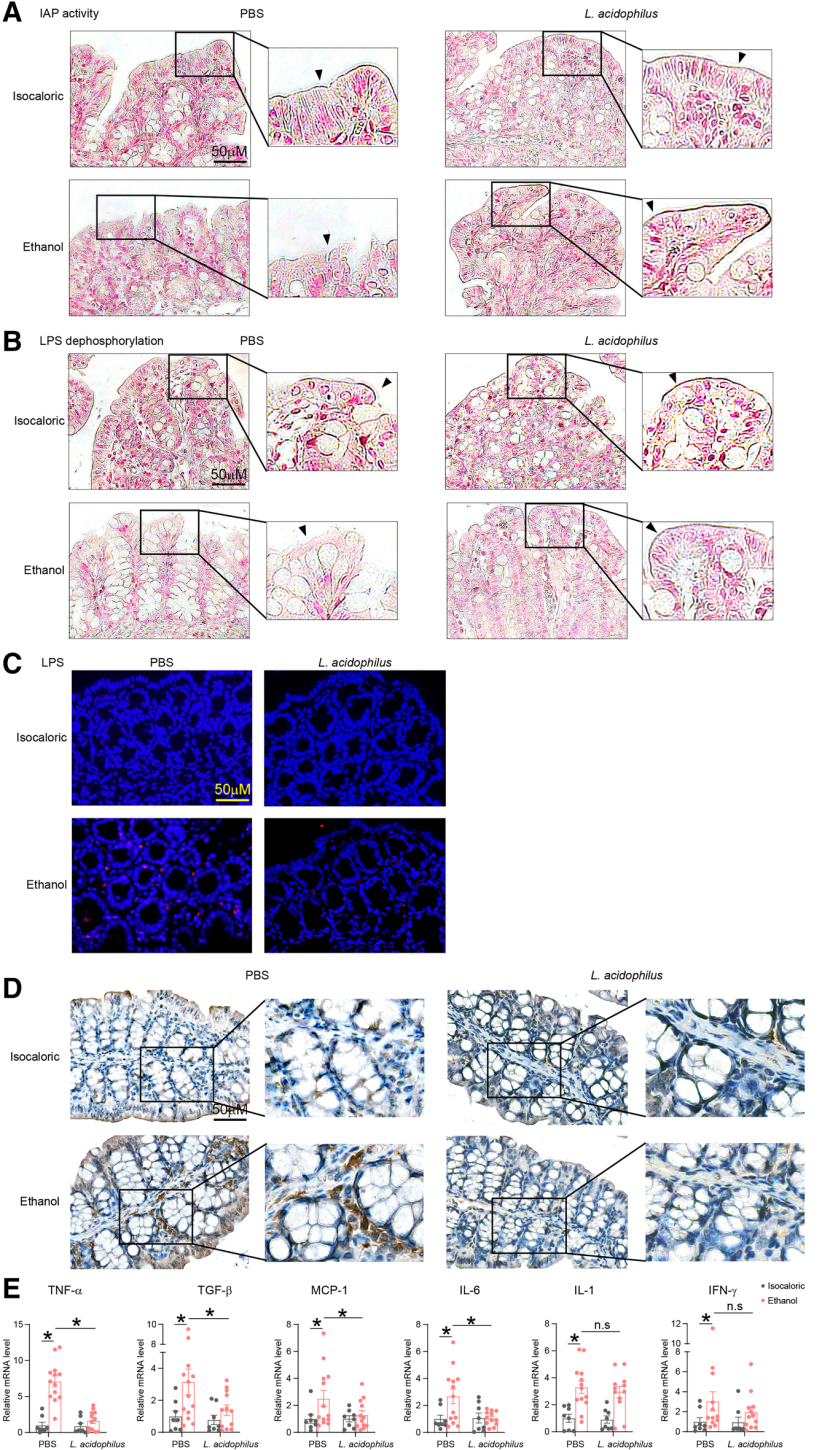
*L. acidophilus* supplementation restored IAP activity and prevented colonic inflammatory injury. **A**, **B** IAP activity (**A**) and LPS dephosphorylation **B** of ALD mice measured by Modified Gomori calcium-cobalt method were recovered by *L. acidophilus* treatment*.* **C** Immunofluorescence assay of LPS to evaluate colonic bacterial invasion in ALD and control mice with and without *L. acidophilus* treatment. **D** F4/80 immunohistochemical staining to evaluate colonic macrophag invasion in ALD and control mice with and without *L. acidophilus* treatment. **E** Expression of inflammatory cytokines measured by PCR of colonic tissues from ALD and control mice with and without *L. acidophilus* treatment. *p value < 0.05

To further elucidate the effect of *L. acidophilus* on the colonic inflammatory response, colonic IAP activity and LPS dephosphorylation levels were measured. Ethanol consumption decreased IAP activity and the level of LPS dephosphorylation, while *L. acidophilus* supplementation could recover IAP activity and LPS dephosphorylation (Fig. 7A, B). Furthermore, bacterial invasion decreased when chronic-binge ethanol-fed mice were supplemented with *L. acidophilus* (Fig. 7C). Given that IAP can prevent inflammation, macrophages infiltration levels and inflammatory factors were measured. Alcohol exposure caused inflammation in the colon, characterized by macrophage infiltration and increased expression of inflammatory factors, compared to controls (Fig. 7D, E). *L. acidophilus* supplementation decreased colonic inflammation by reducing macrophage infiltration levels and the expression levels of the inflammatory factors TNF-α, TGF-β, MCP-1, and IL-6 (Fig. 7D, E).

### *L. acidophilus* supplementation restored the dysfunction of alcohol- associated phenylalanine metabolism

**Fig. 8:**
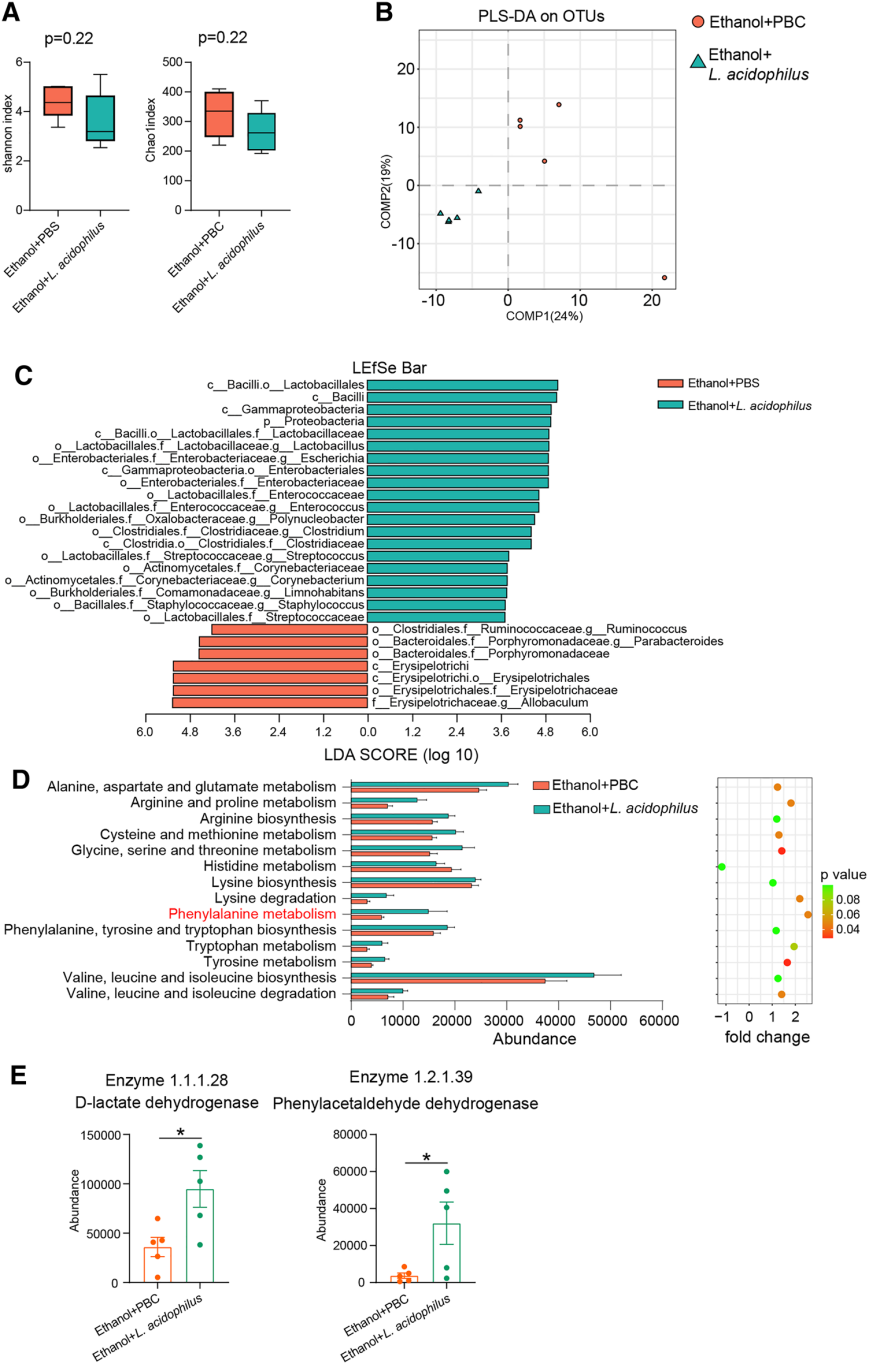
*L. acidophilus* supplementation recovered bacterial phenylalanine metabolism of ALD mice. **A** Shannon and chao indexes measured the alpha diversities of intestinal bacteria of ALD mice with and without *L. acidophilus* supplementation. **B** PLS-DA revealed the beta diversity of intestinal bacteria. **C** Significantly different bacterial taxa examined by LEfSe analysis between ALD mice with and without *L. acidophilus* supplementation. Alcohol exposed and control groups. **D** KEEG module abundances of amino acid metabolism that predicted by PICRUSt between ALD mice with and without *L. acidophilus* supplementation. **E** Comparison of the abundances of phenylalanine metabolizing enzymes D-lactate dehydrogenase and phenylacetaldehyde dehydrogenase between ALD mice with and without *L. acidophilus* supplementation. *p value < 0.05

16S rDNA sequencing of the stool was performed to determine the effect of *L. acidophilus* supplementation on the intestinal microbiota in alcohol-fed mice. As shown in Fig. 8A, the bacterial diversity calculated using the Shannon and Chao indexes was similar in ethanol feeding mice with or without *L. acidophilus* supplementation. The PLS-DA analysis showed that mice fed ethanol with or without *L. acidophilus* supplementation were clustered differently (Fig. 8B). Some bacteria classifications were significantly changed in the ethanol-fed mice with *L. acidophilus* supplementation, compared to mice without *L. acidophilus* supplementation (Fig. 8C, Additional file 1: Figure S2.). Typically, the abundance of the *Lactobacillus* genus (*Lactobacillaceae* family) increased, while the abundance of the *Parabacteroides* genus (*Porphyromonadaceae* family) decreased after *L. acidophilus* supplementation. Phenylalanine metabolism and the abundances of bacterial D-lactate dehydrogenase and phenylacetaldehyde dehydrogenase were increased significantly in mice supplemented with *L. acidophilus*, compared to mice not supplemented with *L. acidophilus* (Fig. 8D, E)*.*

**Fig. 9:**
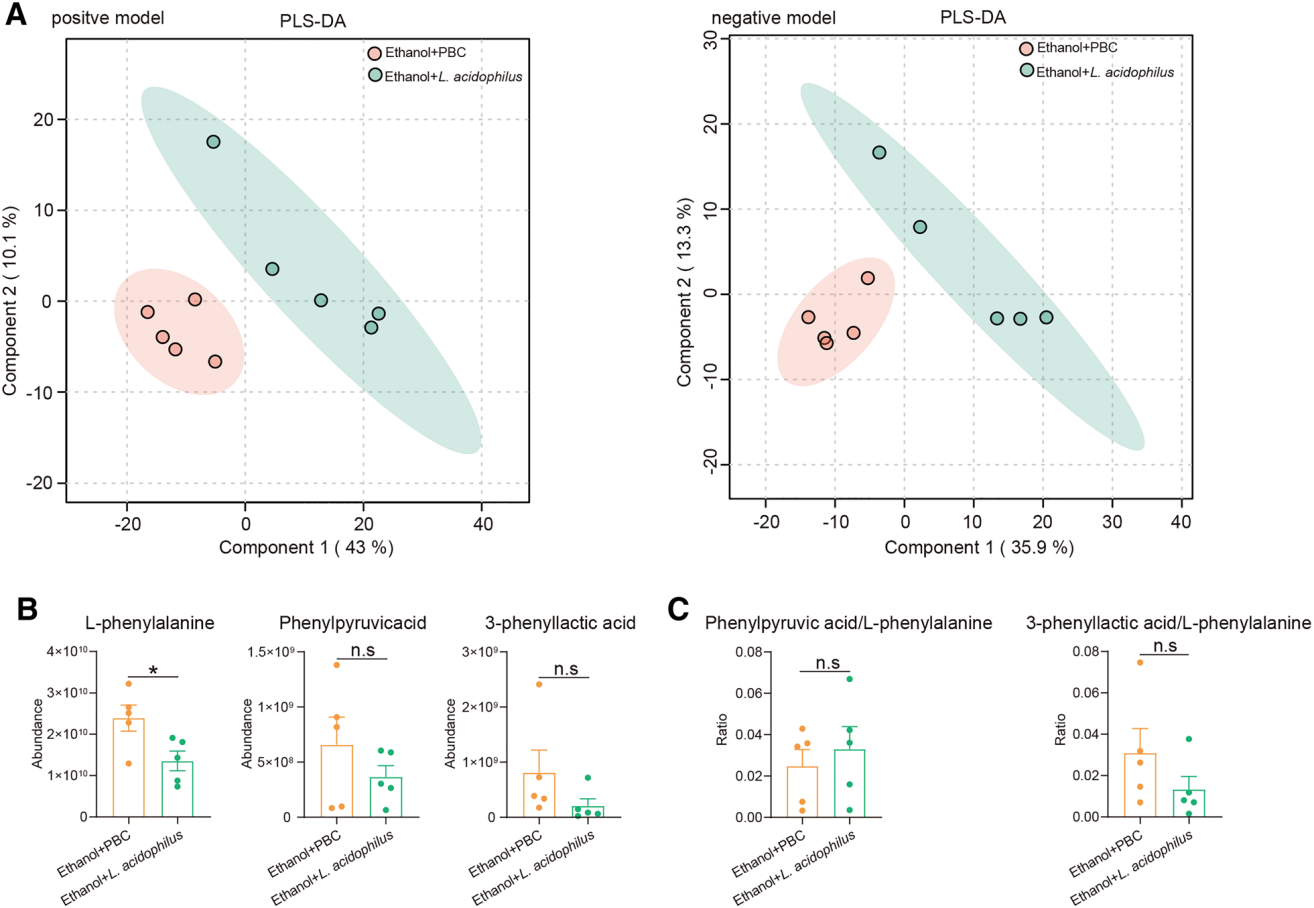
*L. acidophilus* supplementation decreased intestinal phenylalanine of ALD mice. **A** PLS-DA showed that there were significant differences in intestinal metabolites of ALD mice with and without *L. acidophilus* supplementation. **B** Comparison of the abundances of L-phenylalanine, phenylpyruvic acid and 3-phenyllactic acid between ALD mice with and without *L. acidophilus* supplementation. **C** Comparison of the ratios of phenylpyruvic acid to L-phenylalanine and and 3-phenyllactic acid to L-phenylalanine between ALD mice with and without *L. acidophilus* supplementation. n.s, no significant. *p value < 0.05

The fecal metabolites of mice fed ethanol and supplemented with *L. acidophilus* were different from those of mice without *L. acidophilus* supplementation (Fig. 9A). The intestinal phenylalanine level decreased after the alcohol-fed mice were treated with *L. acidophilus* (Fig. 9B), but did not show any differences in the level of phenylpyruvate and 3-phenyllactic acid. And the ratios of phenylpyruvate to L-phenylalanine and 3-phenyllactic acid to L-phenylalanine were same between alcohol-fed mice with and without *L. acidophilus* supplementation (Fig. 9C).

## Discussion

Phenylalanine, an essential amino acid, is converted to tyrosine by phenylalanine hydroxylase in the liver. Subsequently, it is further metabolized into neurotransmitters, such as dopamine (DA), norepinephrine (NE), adrenaline (E), and melanin, which regulate the metabolism of glucose and fat in the human body [32]. In vitro, phenylalanine inhibits hepatocyte glycolysis but does not affect mitochondrial respiration by inhibiting pyruvate kinase and hexokinase, which are key enzymes of the glycolytic pathway [33]. There was a significant increase in intestinal phenylalanine level in patients with colorectal intramucosal carcinomas, compared with healthy controls [34]. However, in ALD, changes in intestinal phenylalanine and its metabolism by bacteria have been not reported. We found that mice exposed to alcohol had a higher level of intestinal phenylalanine and a lower abundance of bacterial enzymes that metabolize phenylalanine, compared to the control mice.

IAP has been found mediating interactions between microorganism communities and the host. IAP dephosphorylates LPS and other pathogen-associated molecular patterns, reducing local intestinal inflammation [35] and helping maintain gut barrier function [29, 36]. IAP supplementation has been reported to be able to ameliorate experimental colitis [37], reverse metabolic syndrome [38], prevent liver fibrosis [21], and attenuate alcohol-induced hepatosteatosis [20]. Decreased IAP expression is associated with impaired barrier function. We found supplement with phenylalanine, a known inhibitor of IAP, exacerbated intestinal barrier function damage of chronic–binge ethanol-fed mice.

Bacterial metabolites of phenylalanine also regulate intestinal and liver homeostasis. Phenyllactic acid (PLA) is an attractive organic acid with a broad antibacterial and antifungal spectrum [26]. A previous study found that the excretion rate of phenyllactic acid in the urine of C57BL/6N mice exposed to alcohol for 3 months had increased significantly, while that of indole-3-lactic acid remained unchanged [39]. Hepatocellular carcinoma patients had significantly higher levels of 3-phenyllactic acid in the portal vein and cancerous tissues than healthy controls, which can be associated with impaired liver function and poor survival [40]. Phenylacetic acid (PAA) triggered hepatic steatosis and increased branched-chain amino acid utilization [41]. In healthy individuals, fecal PAA was found to be negatively associated with the *Clostridium XIVa cluster* and positively with *Lactobacillus* [42]. Individuals with high levels of fecal phenylpropionate had elevated serum levels of TGF-β, IL-17, IL-8, malondialdehyde and C-reactive protein, and were in a low pro-oxidation and pro-inflammatory state [42]. However, changes in these metabolites in the circulatory system and liver of alcohol-fed mice and control mice were not measured in our study. The direct role of phenylalanine metabolites in alcohol-induced liver injury deserves further exploration.

Probiotic *Lactobacilli* has been found to ameliorate ethanol-induced hepatic damage via several ways [43]. *Lactobacillus rhamnosus GG* treatment recovered intestinal barrier functions of ALD mice through increasing tight junction protein expressions [44, 45]. *Lactobacillus rhamnosus GG* culture supernatant restored alcohol-induced intestinal barrier dysfunction by promoting HIF signaling [46]. Long time *Lactobacillus rhamnosus GG* supplementation prevented alcohol-induced colonic dysbiosis [47]. *Lactobacillus plantarum* pretreatment relieved alcohol-induced subacute liver injury by reducing inflammation and enhancing antioxidative response of liver [48, 49]. We found that *Lactobacillus* abundance was negatively correlated with the level of phenylalanine. *L. acidophilus* supplementation alleviated alcohol-induced liver damage, restored IAP activity of colonic epithelial cells, and improved intestinal barrier function. *Lactobacillus* is an important genus that metabolizes phenylalanine, as it stimulates the expression of lactate dehydrogenase, which is responsible for the bioconversion of phenylpyruvate to phenyllactic acid [50]. In fact, *L. acidophilus* supplementation enhance phenylalanine metabolism and decreased the level of phenylalanine in ALD mice, measured by bacteria functional enrichment analysis and metabolomics analysis. Although *Lactobacillus* was obviously negative relation with phenylalanine and its metabolites, other altered amino acids such as valine, leucine and isoleucine are also worthy of future investigation.

## Conclusion

In this study, we reported that alcohol-related intestinal dysbiosis results in bacterial metabolism disorders of phenylalanine. Inhibition of IAP activity of colonic epithelial cells, caused intestinal inflammation and translocation of pathogen-associated molecular patterns, such as bacteria and LPS, leading to liver steatosis and injury. Supplementation with *L. acidophilus* restored phenylalanine metabolism and improved intestinal IAP activity, ultimately improving intestinal barrier function, and offered protection against ethanol-induced liver injury. However, we did not validate our results in a population with ALD, and this can be considered a limitation of this study.

## Supplementary Information

**Additional file 1: Table S1.** Primers used in RT-PCR. **Table S2.** List of Lactobacillus species detected by PCR. **Figure S1.** Pro-inflammatory macrophages infiltration of colon tissues from mice fed ethanol and isocaloric. Colonic tissues were stained with F4/80 (1:100, Abcam) and iNOS (1:100, Arigo) by immunofluorescence according to previous reports [1, 2]. **Figure S2.** Lactobacillus acidophilus in stools of normal controls and alcohol exposed mice with *Lactobacillus* *acidophilus* supplement measured by qPCR. *p<0.05.

## Data Availability

All data relevant to the study are included in the article and supplementary materials. More Data are available on request from the authors.
