# Supplementary material for: Modulating phenylalanine metabolism by L. acidophilus alleviates alcohol-related liver disease through enhancing intestinal barrier function
Source: Cell Biosci. 2023 Feb 4;13:24. doi: 10.1186/s13578-023-00974-z (PMC9899391; doi:10.1186/s13578-023-00974-z)
Supplement: Supplementary file 1 — Additional file 1: Table S1. Primers used in RT-PCR. Table S2. List of Lactobacillus species detected by PCR. Figure S1. Pro-inflammatory macrophages infiltration of colon tissues from mice fed ethanol and isocaloric. Colonic tissues were stained with F4/80 (1:100, Abcam) and iNOS (1:100, Arigo) by immunofluorescence according to previous reports [1, 2]. Figure S2. Lactobacillus acidophilus in stools of normal controls and alcohol exposed mice with Lactobacillus acidophilus supplement measured by qPCR. *p<0.05. [file 13578_2023_974_MOESM1_ESM.docx]

**Additional file 1: Table S1.** Primers used in RT-PCR.

| Gene name | Forward sequence | Reverse sequence |
| --- | --- | --- |
| Mouse 18S | GTAACCCGTTGAACCCCATT | CCATCCAATCGGTAGTAGCG |
| Total bacteria | GTGSTGCAYGGYTGTCGTCA | ACGTCRTCCMCACCTTCCTC |
| Mouse IL-1β | TTGTTGATGTGCTGCTGTGA | TGTGAAATGCCACCTTTTGA |
| Mouse TGF-β | GGCACCATCCATGACATGAACC | CCGCACACAGCAGTTCTTCTCT |
| Mouse IL-6 | CTGCAAGAGACTTCCATCCAG | AGTGGTATAGACAGGTCTGTTGG |
| Mouse TNF-α | CTGAACTTCGGGGTGATCGG | GGCTTGTCACTCGAATTTTGAGA |
| Mouse MCP-1 | TTAAAAACCTGGATCGGAACCAA | GCATTAGCTTCAGATTTACGGGT |
| Mouse IFN-γ | ATGAACGCTACACACTGCATC | CCATCCTTTTGCCAGTTCCTC |

**Additional file 1: Table S2.** List of *Lactobacillus* species detected by PCR.

| *Lactobacillus* species | Number of mice with target bacteria detected by PCR | |
| --- | --- | --- |
|  | Control mice(N=13) | Alcohol-fed mice(N=15) |
| *L. plantarum* | 0 | 1 |
| *L. kunkeei* | 0 | 2 |
| *L. amylophilus* | 1 | 2 |
| *L. sanfranciscensis* | 0 | 2 |
| *L. ruminis* | 2 | 2 |
| *L. parabuchneri* | 0 | 2 |
| *L. acidophilus* | 13 | 0 |
| *L. lindneri* | 0 | 3 |
| *L. amylophilus* | 4 | 1 |
| *L. gallinarum* | 1 | 2 |
| *L. acetotolerans* | 0 | 1 |
| *L. delbrueckii* | 13 | 13 |
| *L. agilis* | 0 | 2 |
| *L. paracasei* | 3 | 4 |
| *L. jensenii* | 0 | 1 |
| *L. brevis* | 8 | 4 |
| *L. sakei* | 0 | 0 |
| *L. johnsonii* | 10 | 8 |
| *L. rhamnosus* | 1 | 2 |
| *L. curvatus* | 0 | 2 |
| *L. salivarius* | 0 | 0 |
| *L. fermentum* | 10 | 6 |
| *L. gasseri* | 1 | 1 |
| *L. reuteri* | 13 | 13 |
| *L. amylovorus* | 2 | 2 |
| *L. heilongjiangensis* | 0 | 2 |
| *L. acidipiscis* | 0 | 0 |
| *L. zymae* | 0 | 3 |
| *L. helveticus* | 0 | 1 |
| *L. coryniformis* | 0 | 2 |
| *L. pentosus* | 0 | 1 |
| *L. crustorum* | 0 | 2 |
| *L. farciminis* | 1 | 2 |
| *L. mucosae* | 0 | 0 |
| *L. buchneri* | 0 | 1 |
| *L. paraplantarum* | 0 | 0 |
| *L. casei* | 0 | 0 |


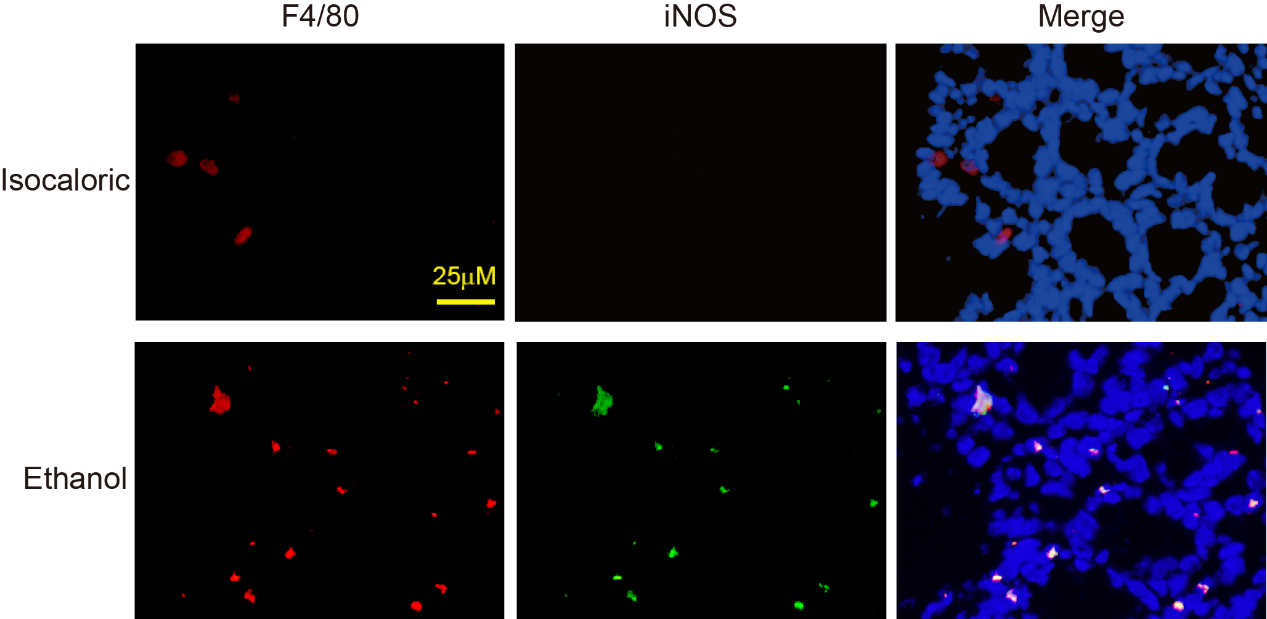


**Additional file 1: Figure S1.** **Pro-inflammatory macrophages infiltration of colon tissues from mice fed ethanol and isocaloric.** Colonic tissues were stained with F4/80 (1:100, Abcam) and iNOS (1:100, Arigo) by immunofluorescence according to previous reports[[1](#_ENREF_6), [2](#_ENREF_7)].


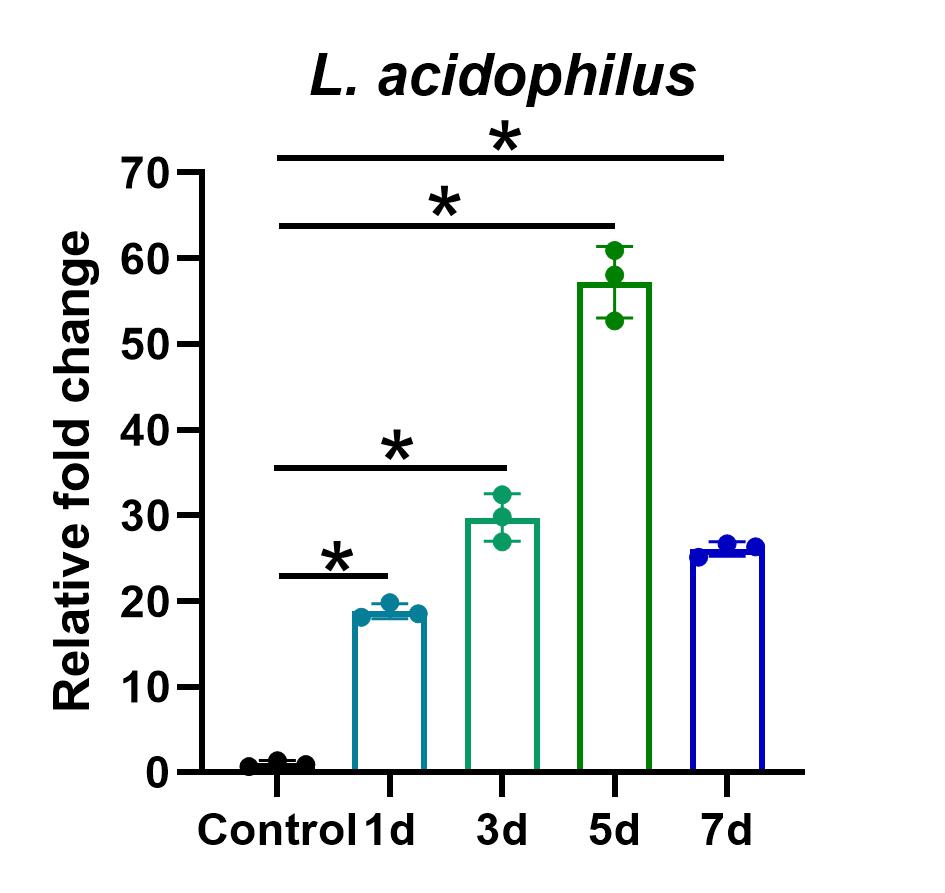


**Additional file 1: Figure S2.** *Lactobacillus acidophilus* in stools of normal controls and alcohol exposed mice with *Lactobacillus acidophilus* supplement measured by qPCR. *p<0.05.

**References**

1. Cao X, Duan L, Hou H, Liu Y, Chen S, Zhang S, Liu Y, Wang C, Qi X, Liu N, Han Z, Zhang D, Han Z-C, Guo Z, Zhao Q, Li Z. IGF-1C hydrogel improves the therapeutic effects of MSCs on colitis in mice through PGE-mediated M2 macrophage polarization. Theranostics. 2020;10:7697-709.

2. Chen Y, Liu Y, Wang Y, Chen X, Wang C, Chen X, Yuan X, Liu L, Yang J, Zhou X. Prevotellaceae produces butyrate to alleviate PD-1/PD-L1 inhibitor-related cardiotoxicity via PPARα-CYP4X1 axis in colonic macrophages. J Exp Clin Cancer Res. 2022;41:1.
